# Towards Streamlined Transparent Data Linkage

**DOI:** 10.23889/ijpds.v9i5.2574

**Published:** 2024-09-18

**Authors:** Michael Edwards, Alexandra Lee, Simon Thompson

**Affiliations:** 1 Swansea University

Linked data is a powerful resource within data analytics and population-level research. However, methods for linkage vary and the choice of approach can impact downstream usage of data by introducing assumptions and biases in resulting links. Selecting stringent linkage methods helps strengthen identified links at risk of missing links; meanwhile, lenient rules or ill-considered comparisons may introduce false positive links. Therefore, the approach is non-trivial, requiring careful selection of preprocessing steps, model development and quality review to ensure suitable outputs, which can require significant human expertise and insight.

Real-world population-scale linkage can benefit from automation and scalability offered within modern data centres, with many tasks eligible for pipelining, such as applying predefined cleaning routines, training defined models, and generating mapping tables. Despite this, there are still pinch points requiring human interaction, such as selecting appropriate linkage fields, blocking rules and comparison methods, and reviewing quality of predictions.

We present an approach to provide scalable automation in linkage pipelines, whilst retaining transparency of the linkage process for downstream users, providing them with a dataset’s life history.

The work output for a given dataset is a versioned catalogue documenting the dataset’s journey, with transparent reporting of data origin, linkage settings, routines, and privacy-preserving quality analysis for inspection. This gives researchers insight into how it may affect their data and provides confidence in data usage. These insights also work in both directions, allowing users to provide feedback and iteratively refine linkage approaches.

